# Recent developments in detection and enumeration of waterborne bacteria: a retrospective minireview

**DOI:** 10.1002/mbo3.383

**Published:** 2016-07-10

**Authors:** Rehan A. Deshmukh, Kopal Joshi, Sunil Bhand, Utpal Roy

**Affiliations:** ^1^ Department of Biological SciencesBirla Institute of Technology and Science, Pilani‐K.K. Birla Goa CampusNH17B BypassZuarinagarGoa403726India; ^2^ Biosensor LabDepartment of ChemistryBirla Institute of Technology and Science, Pilani‐K.K. Birla Goa CampusNH17B BypassZuarinagarGoa403726India

**Keywords:** Bacteria, biosensor, detection, NGS, PCR, rapid, waterborne

## Abstract

Waterborne diseases have emerged as global health problems and their rapid and sensitive detection in environmental water samples is of great importance. Bacterial identification and enumeration in water samples is significant as it helps to maintain safe drinking water for public consumption. Culture‐based methods are laborious, time‐consuming, and yield false‐positive results, whereas viable but nonculturable (VBNCs) microorganisms cannot be recovered. Hence, numerous methods have been developed for rapid detection and quantification of waterborne pathogenic bacteria in water. These rapid methods can be classified into nucleic acid‐based, immunology‐based, and biosensor‐based detection methods. This review summarizes the principle and current state of rapid methods for the monitoring and detection of waterborne bacterial pathogens. Rapid methods outlined are polymerase chain reaction (PCR), digital droplet PCR, real‐time PCR, multiplex PCR, DNA microarray, Next‐generation sequencing (pyrosequencing, Illumina technology and genomics), and fluorescence in situ hybridization that are categorized as nucleic acid‐based methods. Enzyme‐linked immunosorbent assay (ELISA) and immunofluorescence are classified into immunology‐based methods. Optical, electrochemical, and mass‐based biosensors are grouped into biosensor‐based methods. Overall, these methods are sensitive, specific, time‐effective, and important in prevention and diagnosis of waterborne bacterial diseases.

## Introduction

Waterborne diseases have become a major public health issue globally, which affect more than half the population of the developing world. Approximately one billion people depend on contaminated water sources amounting to 2.2 million annual deaths caused predominantly by diarrheal diseases (Miagostovich et al. [Link]) to which World Health Organization (WHO) estimates around 4% of the global disease burden (WHO 2011). UNICEF (2010) survey estimates that the children under the age of five are more prone to diarrheal diseases which account for more than 90% of annual deaths with about 5000 children die per day. Waterborne diseases are not only the problems of developing nations but also a great challenge to the developing nations. Water quality for potable and recreational purposes has been exclusively evaluated based on the culture‐dependent enumeration and detection of fecal indicator bacteria (FIB), for example, total coliforms, *Escherichia coli,* or *Enterococci*, a strategy practiced for decades as a “gold standard” in the assessment of microbial safety of water (Figueras and Borrego 2010). Presence of FIBs preferably *E. coli* and *Enterococcus* at high concentrations in the freshwater serves as proxies (i.e., measurable quantities associated with unknown agents that directly mediate or indicate waterborne risks such as pathogens, biotoxins, and chemicals) for associated pathogens and has been linked to the onset of waterborne diseases in exposed populations (Haile et al. 1999; Colford et al. 2007; Harwood et al. 2014). In India, the number of cases of acute gastroenteritis caused by consumption of contaminated water and poor sanitation have been estimated to be approximately 10.87 million every year. Most of the outbreaks resulted from bacterial and viral pathogens, for example *Escherichia coli, Salmonella* spp*., Shigella* spp., and hepatitis viruses A and B (Bureau of Indian standards 2012).

Etiological agents of most waterborne diseases are broadly categorized into: bacteria, viruses, and parasites (protozoans and helminths). Waterborne bacteria listed in Table 1 adapted from World Health Organization (WHO) (2011) are capable of causing severe diseases which persist in water for a long period. Most microbes in treated drinking water are relatively harmless; however, outbreaks of disease associated with such treated water samples may arise due to compromised water treatment (Howe et al. 2002). Pathogens that withstand disinfection process tend to persist in water distribution pipelines as biofilms which as a result lead to dissemination to end users by the process of sloughing (Figueras and Borrego 2010). Untreated well water also poses great risk, where pathogens such as *Legionella* and *Campylobacter* are the leading well waterborne etiological agents in U.S. (Yoder et al. 2011). As per the guidelines of U.S. environmental Protection agency (USEPA) (2009) and World Health Organization (WHO) (2011), there should hardly be any presence of total coliforms (TC) and *E. coli* in 100 mL of potable water sample. Water is mostly contaminated by feces from infected people and animals which lead to contamination of reservoirs and wells through runoff and permeation into the groundwater (Bosch 1998; Connelly and Baeumner 2012). Moreover, seasonal changes may play an important role in contamination of water sources via runoff from either heavy rainfall or flooding (Ramírez‐Castillo et al. 2015). However, the overall mortality and morbidity caused by intake of contaminated water is high enough and requires to be monitored by enhancing the sanitation of potable water (Pandey et al. 2014). Waterborne pathogens which persist in the environment for a long duration eventually get diluted to low numbers creating an obstacle for their detection and enumeration. Thus, detection of waterborne microbes plays a significant role in monitoring water safety and sanitation mitigating threats that might lead to contamination of water sources. This review deals with the recent developments for the detection of waterborne bacteria focusing significantly on molecular methods of detection and enumeration, their principle, advantages, and limitations.

**Table 1:** List of relevant waterborne bacterial pathogens as given by World Health Organization (WHO)^a^

Pathogen	Disease	Relative infectivity	Persistence in water^b^	Resistance to chlorine^c^
*Burkholderia pseudomallei*	Melioidosis	Low	May multiply	Low
*Campylobacter jejuni, C. coli*	Gastroenteritis	Moderate	Moderate	Low
*E. coli* – pathogenic	Gastroenteritis	Low	Moderate	Low
*E. coli* O157:H7	Gastroenteritis, hemolytic uremia	High	Moderate	Low
*Legionella* spp.	Legionnaires' disease	Moderate	May multiply	Low
Non‐tuberculous mycobacteria	Pulmonary disease, skin infection	Low	May multiply	High
*Pseudomonas aeruginosa*	Pulmonary disease, skin infection^d^	Low	May multiply	Moderate
*Salmonella* Typhi	Typhoid fever	Low	Moderate	Low
*Salmonella enterica*	Salmonellosis	Low	May multiply	Low
*Shigella* spp.	Shigellosis	High	Short	Low
*Vibrio cholerae*	Cholera	Low	Bioaccumulates	Low
*Yersinia enterocolitica*	Gastroenteritis	Low	Long	Low

a Adapted from WHO Guidelines for drinking water Quality (2011).

b Detection period for infective stage in water at 20°C: short, up to 1 week; moderate, 1 week to 1 month; long, over 1 month.

c Infective stage is freely suspended in water treated at conventional doses and pH between 7 and 8. Low means 99% inactivation at 20°C generally in <1 min, moderate 1–30 min and high >30 min. However, an organism which grows in biofilms, for example *Pseudomonas aeruginosa*, resists chlorination.

d immunocompromised individuals.

## Surveillance for Waterborne Disease Outbreak

Water management and sanitation advancements are the basic requirements for human survival around the world at present which have substantially reduced waterborne diseases in the developed nations; however, the scenario is equally dismal in the developing nations. To define waterborne disease outbreak, two or more persons must be reported to be affected epidemiologically by time, location of water exposure and the case illness characteristics must implicate water as the probable source of outbreak (Cutler and Miller 2005). In US, 32 drinking water‐associated outbreaks were reported from 2011–2012 accounting for 431 cases of illness, 102 hospitalizations, and 14 deaths. A total of 66% of outbreaks and 26% of illness were caused by *Legionella*, whereas the viruses and non‐*Legionella* bacteria accounted for 16% of outbreaks and 53% of illness. The outbreaks by non‐*Legionella* bacteria resulted in 90 cases, of which 62% were caused by Shiga toxin‐producing *E. coli* and 24% by *Shigella sonnei* (Beer et al. 2015). For recreational water, 32 states in US reported 90 recreational water‐associated outbreaks to Centers for Disease Control and Prevention's (CDC) Waterborne Disease and Outbreak Surveillance System (WBDOSS) *via* the National Outbreak Reporting System (NORS) from 2011 to 2012. The report revealed 90 outbreaks which were responsible for at least 1788 cases, 95 hospitalizations, and one death. A total of 77% outbreaks were associated with the treated recreational water and 52% were caused by *Cryptosporidium*, whereas untreated recreational water resulted in 23% outbreaks and 33% were caused by *E. coli* O157:H7 or *E. coli* O111. National outbreak and laboratory data (e.g., molecular typing of *Cryptosporidium*) was suggested to be used for the prevention and control of outbreaks associated with recreational water (Hlavsa et al. 2015). European Centre for Disease Prevention and Control (ECDC) reported the outbreaks of waterborne diseases such as campylobacteriosis, cholera, cryptosporidiosis, leptospirosis, listeriosis, salmonellosis, and yersiniosis. In 2012, 217261 confirmed cases of campylobacteriosis, 9591 cases of cryptosporidiosis, and 92438 cases of salmonellosis and 5748 cases of Shiga toxin/verocytotoxin‐producing *E. coli* (STEC/VTEC) infection were reported from the European Union (EU). Outbreaks resulting from campylobacteriosis, salmonellosis, shigellosis, and *E. coli* infection were rather prevalent in more than 25 EU states, whereas cryptosporidiosis was reported mainly from four EU states mainly from the United Kingdom (68%), Ireland (6%), Belgium (5%), and Germany (14%) (ECDC 2014). It is interesting to note that in developing nations, for instance India, 10 million cases of diarrhea, more than 7.2 lakh typhoid cases, and 1.5 lakh viral hepatitis cases are reported every year. Most of the cases are associated with unclean water supply and improper sanitation (Bureau of Indian standards 2012; Saxena et al. 2015). Nevertheless, outbreak surveillance data should not be used to estimate the total number of cases of waterborne diseases mainly because the disease outbreak data might be incomplete as the methods used to strengthen the evidence implicating water as a source of infection vary by nations and localities (Beer et al. 2015).

## Culture‐Based Methods

Numerous conventional culture‐based methods such as multiple‐tube fermentation (MTF) technique, membrane filtration technique, and standard bacterial cell culture methods have been used for cultivation, enumeration, and identification of fecal indicators and pathogens. In MTF, series of lactose broth or lauryl tryptose broth tubes are inoculated with appropriate decimal dilutions of water sample. A positive presumptive test shows production of acid and gas in the medium after 48 h of incubation at 35^**°**^C. Subsequently, tubes with positive presumptive test are subject to confirmation test. A positive confirmation tube is accompanied by formation of gas in a brilliant green lactose bile broth within 48 h at 35°C. Finally, the results of MTF are interpreted in terms of most probable number (MPN) of microorganisms present in the water sample. Hence, MPN provides statistical estimate of mean of total coliforms present in the sample (Evans et al. 1981; Seidler et al. 1981; Rompre et al. 2002). There are factors which limit the efficiency of detection of coliforms such as interference by non‐coliform bacteria (Means and Olson 1981; Seidler et al. 1981) and the inhibitory nature of media (McFeters et al. 1982).

Membrane filtration technique consists of filtering 100 mL of water sample on a sterile 0.45 *μ*m filter, which retains bacteria followed by incubating filter on a selective medium allowing the growth of bacteria (Rice et al. 1987). Examples of selective media include the m‐Endo‐type medium and Tergitol‐TTC medium (Rompre et al. 2002). However, major limitations of the culture‐based enumeration methods suffer from lack of discrimination between the targeted and endogenous microbiota of the environmental sample, false‐positive counts, laborious and time‐consuming protocols, and occasional failure in the detection of viable but noncultivable (VBNC) cells (Sohier et al. 2014). VBNC state is a crucial adaptive mechanism found in nonspore‐forming bacteria, and therefore, it is important to investigate the association of bacterial pathogens under VBNC state and the water/foodborne outbreaks.

Microbial culture is one of the primary steps in microbiology and there are many standard microbial culture methods which enable growth and enumeration of viable bacteria (Talaro et al. 2002). However, there are some microorganisms such as *E. coli* and *Vibrio cholerae*, which could enter a distinct physiological state known as viable but nonculturable (VBNC) state (Xu et al. 1982). VBNC cells are living cells, however, they lose their ability to grow on microbiological media which normally allow their growth (Oliver et al. 2005). Cells with damaged membranes which cannot retain chromosomal and plasmid DNA are considered as dead, whereas VBNC cells have an intact cell membrane with undamaged genetic material (Heidelberg et al. 1997; Cook and Bolster 2007). VBNC cells are metabolically active and carry out the process of respiration (Besnard et al. 2002) while the dead cells are metabolically inactive. For example, in *Listeria monocytogenes,* ATP level was found to be high 1 year after assuming the VBNC state (Lindbäck et al. 2009). Many bacteria enter VBNC sate under nutritionally deficient conditions and low temperatures (Biosca et al. 1996; Du et al. 2007) indicating an adaptive strategy for long‐term survival of bacteria under unfavorable environmental conditions (Ducret et al. 2014). For example, *Vibrio parahaemolyticus* is more resistant to hostile environment with low pH when they assume VBNC state (Wong and Wang 2004). During microbiological examination of environmental sample, the presence of VBNC cells would quite obviously tend to suppress the total number of viable bacteria in a sample in terms of the CFU count method. Moreover, if all bacteria enter VBNC state, the sample may be declared as germ‐free because of nondetection (Li et al. 2014). Bacteria causing infections in some cases are underestimated or undetected in quality control samples from food sector or water distribution systems, or clinical samples may usher severe risks to the public health (Pai et al. 2000) which may subsequently lead to emergence of disease in convalescent patients (Rivers and Steck 2001). And also the conversion of avirulent bacteria to virulent state with the revival of the bacteria from the VBNC state bears the potential for serious threat to the public health (Du et al. 2007). Therefore, it is of paramount importance to employ an appropriate detection and quantification methods to differentiate between viable, VBNC and dead cells.

Colilert test, which was proposed as an alternative test to conventional water quality monitoring is based on *β*‐galactosidase activity using a chromogenic substrate Indoxyl‐*β*‐glucuronide (IBDG) for *E. coli* detection and enzymatic cleavage of the fluorogenic substrate, 4‐methylumbelliferyl‐*β*‐galactopyranoside (MUG) to detect total coliforms (Bej et al. 1991a,b). Specialized medium containing IBDG and MUG has been commercialized in the market, for example, MI agar (BDH) facilitating detection of *E. coli* and total coliforms in 100 mL of potable water sample within 24 h of incubation at 37^°^C. The MI medium supplemented with cefsulodin further inhibits the growth of gram‐positive and some non‐coliform gram‐negative bacteria which may cause false‐positive results (Chao et al. 2004). Besides this, the Enterolert system was designed to detect enterococci in 100 mL water sample in 24 h which uses 4‐methylumbelliferyl‐*β*‐glucoside. This compound when hydrolyzed by enterococcal‐*β*‐glucosidase releases 4‐methylumbelliferone which fluoresces under UV_365_ lamp. Hence, the use of conventional methods needs be supported immensely by more rapid and less laborious molecular methods of detection and enumeration.

## Nucleic Acid‐Based Methods

Nucleic acid‐based methods involve detection of specific DNA or RNA sequences in the target pathogens. This is accomplished by hybridization of target nucleic acid sequence to a synthetic oligonucleotide (Ramírez‐Castillo et al. 2015). Pathogens including bacteria, viruses, protozoans, and helminthes which were reported as the leading etiological agents of waterborne diseases (Straub and Chandler 2003; Cabral 2010). These pathogens and their toxin‐producing genes could be detected by nucleic acid‐based methods (Rompre et al. 2002). Nucleic acid‐based methods are rapid and time efficient and can be performed without culturing the pathogens. The nucleic acid‐based methods described are polymerase chain reaction (PCR), droplet digital PCR, multiplex polymerase chain reaction (mPCR), real‐time or quantitative polymerase chain reaction (qPCR), microarrays, pyrosequencing, and fluorescence in situ hybridization (FISH).

## Rapid DNA Extraction Methods

**Figure 1:**
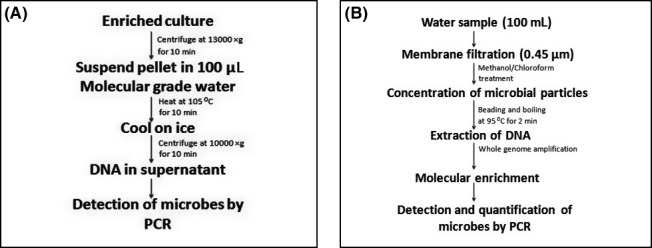
Schematic representations of rapid DNA extraction methods. (**A**) DNA purification from enriched culture (Trkov and Avgustin, 2003). (B) CRENAME method for DNA extraction from waterborne bacteria directly from environmental water sample (Maheux et al. 2011).

Trkov and Avgustin (2003) reported boiling and centrifugation method for extraction of DNA. A quantity of 1 mL of enriched culture is centrifuged at 13000*g* for 10 min. Pellet obtained is resuspended in 100 *μ*L of molecular‐grade water and heated at 105^°^C in dry bath for 10 min, which is then cooled on ice and centrifuged at 10000*g* for 10 min. Supernatant obtained is directly used for polymerase chain reaction (Fig. 1A). However, environmental samples such as water contains very low numbers of microbes within a diverse group of microflora. Methods for DNA extraction from such environmental samples are required to be developed for rapid detection and enumeration of waterborne bacteria. Maheux et al. (2011) developed rapid concentration and recovery of microbial particles, extraction of nucleic acids and molecular enrichment (CRENAME) method for DNA extraction from environmental water samples using methanol and chloroform treatment (Fig. 1B). CRENAME method (Maheux et al. 2011) which involves, (1) method for concentration and recovery of waterborne microbes in 100 mL of water sample passed thorough 0.45‐*μ*m membrane filter. Microorganisms impinged on membrane filter are exposed to methanol and chloroform treatment, (2) nucleic acid extraction procedure by boiling at 95^°^C for 2 min, and (3) molecular enrichment by whole‐genome amplification (WGA) using Illustra GenomPhi DNA amplification kit (GE Healthcare). WGA reaction involves incubation of crude cell extract at 95^°^C for 3 min and cooling on ice for a minimum of 3 min. Subsequently, crude cell extract is mixed with Ф29 DNA polymerase (GenomiPhi DNA amplification kit) and incubated for 3 h at 30^°^C. Enzymatic reaction is stopped by 10 min incubation at 65^°^C and WGA‐amplified product is then directly used for qPCR. This method enabled the detection of 1.8 CFU of *E. coli* in 100 mL of environmental water sample within a span of 5 h.

Concentration is a preliminary step for detection of many waterborne pathogens. However, this step seems troublesome because the recovery efficiencies using conventional methods, for instance, filtration, are low and often variable which in turn may lead to false‐negative results (Zuckerman and Tzipori 2006). Moreover, analysis of water sample largely depends on the nature and volume of water and the type of filter used for the assay. About 500 to 1000 mL volume of water sample and filter size ranging from 0.22 to 0.45 *μ*m have been employed for detection and quantification of waterborne pathogens (Maheux et al. 2011). Centrifugation method such as continuous separation channel centrifugation is one of the effective methods for concentrating waterborne pathogens which can simultaneously concentrate multiple pathogens as small as 1 *μ*m with reproducible efficiencies in a variety of water samples.Borchardt and Spencer (2002) reported spiking of *Cryptosporidium parvum* oocysts, *Giardia lamblia* cysts, *Encephalitozoon intestinalis* spores, and *E. coli* in different water matrices at densities ranging from 5 to 10000 organisms L^−1^ and recovered using continuous separation channel centrifugation. The system could be operated at 2000, 2500, and 3000 g and flow rates of 0.5, 1, and 1.4 L min^−1^, respectively. The highest recovery, 94%, was obtained at 2500 g with a flow rate of 1 L min^−1^. Many rapid DNA extraction kits have been commercialized in the market such as Purgene cell and tissue kit (Gentra) and BD Diagnostics‐GeneOhm Rapid Lysis kit (BD Diagnostics) (Li and Chen 2012; Maheux et al. 2014). van Tongeren et al. (2011) reported comparative analysis of three rapid and easy bacterial DNA extraction methods for use with real‐time polymerase chain reaction mainly QIAamp^®^ DNA Mini Kit (QIAGEN, Germany), Reischl et al.'s (1994) method and FTA^®^Elute (GE Healthcare, United Kingdom). FTA^®^ Elute demonstrated the highest DNA extraction efficiency of 76.9% for *E. coli* and 108.9% for *Staphylococcus aureus*. The Reischl et al. (1994) method and QIAamp^®^ DNA Mini Kit were noted to inhibit *E. coli* qPCR assay with a 10‐fold decrease of detectable DNA. However, none of the three methods inhibited the *S. aureus* qPCR assay. Thus, FTA^®^ Elute was the only method found to be suitable in terms of efficiency of DNA extraction, rapidity, ease of use, toxicity, transport, and storage.

## Polymerase Chain Reaction

PCR is the most commonly employed molecular‐based method for the detection of waterborne pathogens. PCR allows the detection of a single bacterial pathogen which is present in water by targeting specific DNA sequence (Maheux et al. 2011). In PCR, a specific DNA sequence is amplified in a cyclic three‐step process (Mandal et al. 2011) involving denaturation, annealing, and extension. Denaturation is the unwinding of DNA duplex to single‐stranded DNA. Following this, the two specific primers anneal to DNA strands. Finally, thermostable DNA polymerase carries out the polymerization process where the primers complementary to the single‐stranded DNA are extended in the presence of deoxyribonucleotides and suitable ions necessary for the activity of DNA polymerase. Thus, PCR cycling significantly increases the specificity of detection of target DNA in low numbers of microorganisms in an environmental sample (Bej et al. 1990; Khan et al. 2007; Maheux et al. 2013). The PCR amplified products are detected by agarose gel electrophoresis after staining with ethidium bromide (Khan et al. 2007). PCR has been employed for the detection of various waterborne pathogens like *E. coli,* enterotoxigenic *E. coli* (ETEC), and spores of *Clostridium perfringens* (Tsen et al. 1998; Ram et al. 2008; Maheux et al. 2013). Bej et al. (1991a,b) reported *lacZ* gene for the detection of coliforms in water. Later, Fricker and Fricker (1994) used the same primer set as Bej et al. (1991a,b) on 324 cultured coliform strains and concluded that *lacZ* gene could not discriminate some of *Hafnia alvei* and *Serratia odorifera* strains. Use of *uidA* (encodes *β*‐D‐glucuronidase) gene was then envisaged for the detection of *E. coli* (Tsai et al. 1993). The regulatory region (*uidR*) and the *uidA* gene were used successfully and it was found that the primer set was specific for *E. coli* and *Shigella* spp. (Juck et al. 1996; Iqbal et al. 1997).

## Droplet Digital PCR (ddPCR)

Droplet digital PCR is a new technique where PCR products labeled with digoxigenin‐UTP (DIG‐dUTP) and anti‐DIG Fab' fragments conjugated with fluorescent dye are used for detection by epifluorescence microscopy. The distribution of the reaction mix into separate droplets *via* microfluidics is subjected to thermal cycling followed by individual screening by fluorescence‐enabled measurement for the target copy number of the DNA without the standard is the strength of this technique (Bhat et al. 2009). The ddPCR allows a direct visualization of the fluorescent amplification products at a single‐cell level and consequently a possible direct enumeration of labeled‐cells. Even though the results show a weak fluorescence intensity signal of targeted cells, the image analysis allows a direct enumeration of target cells (Rompre et al. 2002). Rothrock et al. (2013) used ddPCR for quantification of *Salmonella* spp., *Campylobacter jejuni*, and *Listeria monocytogenes* in environmental water samples. Similarly, the direct enumeration of *E. coli* from freshwater samples can be performed.

## Real‐time or Quantitative PCR (qPCR)

The detection and quantification of target nucleotide sequences with utmost precision and as small as a few copies within few hours has been a dream even a decade ago. However, quantitative real‐time PCR (qPCR) system has proven to be a mighty tool in the field of water quality determination (Girones et al. 2010). Real‐time or quantitative PCR allows evaluating the PCR products amplification by measuring fluorescent signals emitted by specific dual‐labeled probes or intercalating dyes unlike the conventional PCR which requires agarose gel electrophoresis for the detection of PCR products formation. qPCR follows a principle wherein the fluorescence intensity is directly proportional to the amounts of PCR products formed (Rompre et al. 2002; Ramírez‐Castillo et al. 2015). SYBR green, TaqMan probes, and molecular beacons are most commonly used fluorescent systems for qPCR. Numerous protocols have been developed for the detection of waterborne pathogens using the fluorescent systems (Table 2). qPCR is a versatile instrument which can be exploited for many applications such as absolute and relative quantifications of waterborne pathogens. Absolute quantification is used to quantify a gene and express it as an absolute value, for example, copies/mL. Samples with an unknown concentration of gene are amplified against a standard dilution series of specific gene with known concentration. The absolute value for an unknown quantity of a gene is determined by cycle of quantification (Cq) values of unknown sample with respect to those of standards with known quantities (Leong et al. 2007). Relative quantification involves comparison of the levels of two different gene sequences in a single sample, for instance, target gene of interest and a reference gene and the results are expressed as a ratio of these genes (Pfaffl 2001). A sensitive CRENAME method coupled with real‐time PCR was reported for specific detection of *E. coli*/*Shigella* in potable water using TaqMan probe (Maheux et al. 2011). This assay allowed the detection of *E. coli*/*Shigella* as low as 1.8 CFU in 100 mL of potable water. Viability is a major concern for waterborne bacteria because DNA isolated from dead cells can serve as the template for PCR amplification as efficiently as the DNA extracted from viable cells which may lead to overestimating the viable cell numbers in potable water as the DNA molecules can withstand degradation for weeks after the cell death (Way et al. 1993; Sheridan et al. 1998; Vesey et al. 1998; Wang et al. 2009).

**Table 2:** Nucleic acid‐based methods for the detection of waterborne bacteria present in potable water samples

Detection method	Waterborne bacteria	Water matrix	Gene(s) targeted	Limit of detection	Reference
Conventional PCR	Total coliforms	Cultured strains	*lacZ* (326 bp)	1 to 5 CFU	Bej et al. 1990;
	Total coliforms	Cultured strains	*lacZ* (264 bp)	–	Fricker and Fricker 1994;
	*E. coli* and *Shigella* spp.	Cultured strains	*uidR* (154 bp)	–	Fricker and Fricker 1994;
	*E. coli* and *Shigella* spp.(Nested PCR)	Cultured strains	*uidA (486 bp)*	2–0 cells/100 mL	Juck et al. 1996;
	*E. coli* and *Shigella* spp.	Cultured and polluted river water samples	*uidA* (147 bp)*uidR* (154 bp)	–	Iqbal et al. 1997;
	*E. coli* and *Shigella* spp.	Cultured strains	V3 and V6 regions of 16S rRNA (584 bp)	1–2 Cells/100 mL	Tsen et al. 1998;
mPCR	Total coliforms	Cultured strains	*lacZ* (264 bp)	1 cell	Bej et al. 1991a,b;
	*Shigella* spp.*Salmonella* spp.*EHECV. parahaemolyticusP. aeruginosa*	Environmental water samples	*ipaH* (611 bp)*ipaB* (315 bp)*hlyA* (366 bp)16S‐23S rDNA (165 bp)*oprL* (504 bp)	10^2^ CFU10^1^ CFU10^1^ CFU10^2^ CFU10^2^ CFU	Fan et al. 2008;
Real‐time or qPCR	*L. monocytogenes*	Artificial biofilms	*hly* (106 bp)	6 × 10^2^ CFU/cm^2^	Guilbaud et al. 2005;
	*E. coli*	Agricultural watersheds	16S‐23S *rDNA* (450 bp)	10 cell/mL	Khan et al. 2007;
	*E. coli* and *Shigella* spp.	Potable water	*tuf* and TaqMan probe	1.8 CFU/100 mL	Maheux et al. 2011;
	Detection of viable *E. coli*	waste water Biosolids	*uidA* (67 bp) and TaqMan probe	–	Taskin et al. 2011;
	Selective detection of viable *E. coli* O157:H7	Cultured stains	ORF *Z3276* and TaqMan probe	–	Li and Chen 2012;
	*E. coli*,*Enterococcus* spp.*,* and *Salmonella* spp.	Contaminated marine sediments	16S *rDNA*23S *rDNA* and*invA*	20 cells29 cells16 cells	Luna et al. 2012;
	*Clostridium perfringens* spores	Drinking water	*cpa*	–	Maheux et al. 2013;
Microarray	*Bacillus anthracis Brucella abortus,C. botulinum*,*Coxiella burnetii*,* Francisella tularensis, Rickettsia prowazekii,C. perfringens, S. aureus*,* V. cholerae*,* V. alginolyticus*,* Yersinia pestis*	Ocean water and Environmental water spiked with bacteria	Oligonucleotide probes	10 fg for *Bacillus anthracis* DNA,500 fg for *F. tularensis* DNA and 500 fg *Y. pestis* DNA	Wilson et al. (2002)
	*Y. enterocolitica*,*E. coli* and*S. enterica* Typhimurium	Waste water	16S rRNA,cpn60, andwecE gene probes	1 × 10^7^ for *S. typhimurium* cells	Maynard et al. [Link];
	*E. coli* (DNA microarray coupled with qPCR)	Municipal wastewater treatment plant	16S rRNA probe	5 ng of *E. coli* DNA	Lee et al. 2010;
NGS	*B. anthracis*	Bottled water, milk and juice	Virulence markers of plasmid *pXO1* and *pXO2*	6 CFU/mL	Amoako et al. 2013;
	Proteobacteria and Bacteroides	Potable water of nonchlorinated distribution system	*–*	1.3 × 10^5^ cells/mL	Lautenschlager et al. 2013

DNA‐based molecular methods cannot distinguish between viable and membrane‐compromised or extraneous DNA persisting in the environment which in turn contributes to overestimation of pathogens in the water sample (Paul et al. 1989). Two chemicals, propidium monoazide (PMA) and ethidium monoazide (EMA) have been employed to address this problem. PMA permeates only membrane‐compromised cells (Nocker et al. 2007) or binds to extraneous dsDNA in the water sample. PMA has been demonstrated to distinguish live from “dead” cells, extraneous DNA, and cysts, including *Mycobacterium avium* (Nocker et al. 2006), fungal conidia (Vesper et al. 2008), *Cryptosporidium* oocysts (Brescia et al. 2009), and *Bacillus subtilis* spores (Rawsthorne et al. 2009). The photoinducible azide group of PMA allows it to covalently intercalate DNA of dead cells upon exposure to bright light. Subsequently, DNA is rendered insoluble and is lost during genomic DNA purification. In comparision to PMA, EMA has been found to be less efficient, mainly because of its ability to penetrate live cells of some bacterial species (Nocker et al. 2006). Taskin et al. (2011) developed a qPCR assay to selectively quantitate viable *E. coli* with propidium monoazide (PMA) using *uidA* gene. Known quantities of live and dead cell mixtures were used to study the effect of PMA and it was reported that the PMA could inhibit PCR amplification from dead cells with no less than 95% efficiency (Taskin et al. (2011).

Hellein et al. (2012) demonstrated membrane filtration‐based concentration of cultured *Pseudomonas aeruginosa* ATCC 9027 and sewage‐spiked environmental fresh water samples using membrane filtration and subsequently optimized procedure for live–dead discrimination by PMA‐based qPCR. *P. aeruginosa* was grown in Luria‐Bertani (LB) broth with constant shaking for 8–12 h at 37^°^C and finally adjusted to an OD_600_ of 1.0 using LB broth (approximately 10^9^ bacteria/mL). To discriminate live from dead bacteria, fourteen 500 *μ*L aliquots were transferred into 1.5 mL tubes and seven were heat‐killed at 72^°^C for 15 min. Further, six each of live and heat‐killed aliquots were spiked in 100 mL of phosphate buffer saline (PBS), whereas one heat‐killed cell aliquot was grown on LB agar to indicate the loss of viability of cells which was found to be <2 CFU/mL. Six liters of environmental fresh water samples were spiked 1:100 with untreated sewage obtained from local lift stations in USA (Hellein et al. 2012). Three liters of the spiked solution were heat‐killed at 72^°^C for 15 min while the remaining half was maintained unheated (live). The heat‐killed cells were plated on LB agar which did not yield colonies (<2 CFU/mL). A quantity of 100 mL of both live and heat‐killed *P. aeruginosa* and 500 mL of sewage‐spiked fresh water aliquots were filtered through 0.45‐*μ*m, 47‐mm polyethersulfone filters. It was observed that the polyethersulfone filter membranes outperformed polycarbonate membrane filters which allowed much larger volumes of relatively dirty environmental water before clogging and facilitated PMA treatment. However, nitrocellulose membrane filters permitted large volumes of water without membrane clogging although PMA failed to discriminate between live from dead cells trapped on nitrocellulose membrane filters (Hellein et al. 2012). Individual filters were flooded with 300 *μ*L of either 100 *μ*molL^−1^ PMA or PBS in 50 mm Petri dishes and further incubated in dark for 20 min followed by 15 min exposure to 460 nm light and filters were then frozen at −20^°^C till processing. Universal bacterial primers NadF and NadR with probe NadP were employed to amplify 466 bp fragment from the 16S rRNA gene by qPCR as described by Nadkarni et al. (2002). Three different research groups at different locations used local environmental water samples and concluded that PMA greatly reduced amplification of DNA from heat‐killed samples.

However, PMA‐qPCR still requires some improvements in terms of its effectiveness on gram‐positive bacteria (Løvdal et al. 2011; Alvarez et al. 2013), partial resistance to PMA when employing universal bacterial PCR targets and heat‐killing of bacteria or membrane‐compromised cells may be less effective than exposure to isopropanol (Nocker et al. 2006) or hypochlorite (Nocker et al. 2007). Li and Chen (2012) reported selective detection of viable *E. coli* O157:H7 by targeting Z3276 (open reading frame marker). Microfluidic qPCR has been shown to detect pathogens such as *L. monocytogenes*,* V. cholerae*,* V. parahaemolyticus*,* Pseudogulbenkiana* spp., *Salmonella* Typhimurium, *S. flexneri* and *C. perfringens* and *E. coli* at limit of detection of 100 cells/L using TaqMan probes labeled with different fluorophores (Ishii et al. 2014). Real‐time mPCR has been demonstrated by Maheux et al. (2014) targeting *LacZ, WecG*, and 16S rRNA to detect total coliforms and *E. coli* in 100 mL of potable water sample. Results showed that *LacZ, WecG*, and 16S rRNA qPCR assays detected 133 (90.5%), 111 (75.5%), and 146 (99.3%) of the 147 total coliforms strains tested, respectively. Conventional and mPCR are laborious and time consuming as they require detection of PCR products by agarose gel electrophoresis, whereas qPCR allows high‐throughput analysis, low risk of cross contamination, automation, and no post‐PCR detection of amplified products (Luna et al. 2012). Despite the high specificity of qPCR, it poses some limitations where it cannot provide information on the physiological state of the target cells in the environmental samples. Environmental samples, particularly water samples contain humic substances which interferes with the activity of DNA polymerase and some colloidal matters were found to have affinity for DNA (Way et al. 1993; Bustin et al. 2009). As such, there is no universal solution to circumvent these problems. Thus, presence of such substances in environmental samples has the potential to significantly reduce amplification efficiency of qPCR used to detect low numbers of bacteria (Straub 1995). DNA dilution and addition of various substances such as bovine serum albumin, dimethyl sulfoxide, methoxsalen, and internal amplification controls have been proposed to overcome these problems in qPCR. However, these approaches may present some limitations and advantages (Klerks et al. 2004; Shanks et al. 2008; Maheux et al. 2011).

## Multiplex PCR (mPCR)

Multiplex PCR is a more rapid detection assay than the conventional PCR which allows simultaneous detection of multiple gene targets. Primer design is significantly an important step as multiple sets of highly specific primers are used in mPCR assay since primers should have similar annealing temperatures to produce a successful mPCR assay (Omar and Barnard 2014). Other factors which contribute to mPCR assay include primers and PCR buffer concentrations, quantities of DNA template, Taq DNA polymerase, cycling temperatures, and a balanced concentrations of magnesium chloride and deoxyribonucleotides (Markoulatos et al. 2002; Omar and Barnard 2014). Omar and Barnard (2014) reported an mPCR assay to distinguish between pathogenic and commensal *E. coli* from clinical and environmental water sources. The optimized mPCR was developed to study the presence of 11 virulence genes in *E. coli* mainly *eaeA* (intimin)*, stx1* (shiga‐like toxin 1)*, stx2* (shiga‐like toxin 2)*, it* (heat labile enterotoxin)*, st* (heat stable enterotoxin)*, ial* (invasion toxin)*, eagg* (enteroaggregative toxin)*, astA* (EAST1 toxin)*,* and *bfp* (bundle‐forming pili). Furthermore, *mdh* gene (malate dehydrogenase) and *gapdh* gene (glyceraldehyde 3‐phosphate dehydrogenase) were used as controls to evaluate the sensitivity of the method and false‐negative results due to PCR inhibitors. Fan et al. (2008) developed mPCR assay to detect *Enterohaemorrhagic E. coli, Shigella* spp*., Vibrio parahaemolyticus, Pseudomonas aeruginosa,* and *Salmonella* spp. simultaneously in one tube with detection sensitivities of 10^1^, 10^2^, 10^2^, 10^2^, and 10^1^ CFU of one assay, respectively. A similar approach was attempted to detect total coliforms and *E. coli* in potable water samples by targeting *yaiO, uidA*, and *lacZ* genes. PCR amplification *of yaiO* gene was reported to be highly specific compared to *uidA* gene, however, *yaiO* gene amplification could detect some species of *Shigella* (Molina et al. 2015).

## Microarray

Microarrays are powerful genomic technology that are widely used to study gene expression under different cell growth conditions, detect specific mutations in DNA sequences, and characterize microorganisms in the environmental samples (Lee et al. 2008). DNA microarrays are performed with immobilized high‐density nucleic acids (genomic DNA, cDNA, or oligonucleotides) in an ordered two‐dimensional matrix enabling the simultaneous detection of hundreds of genes in a single assay *via* the phenomenon known as nucleic acid hybridization (Zhou 2003; Ramírez‐Castillo et al. 2015). A number of different formats of microarrays have been reported for bacterial detection and understanding microbial communities in the environment having high‐throughput, specificity, and sensitivity. However, microarrays have relatively high cost and may involve nonspecific hybridization resulting in low specificity and sensitivity (Gilbride 2014). Wilson et al. (2002) identified 18 pathogenic microorganisms including bacteria (*Bacillus anthracis, Brucella abortus, Clostridium. botulinum*,* Coxiella burnetii*,* Francisella tularensis, Rickettsia prowazekii, C. perfringens, S. aureus*,* V. cholerae*,* V. alginolyticus*, and *Yersinia pestis*), eukaryotes, and viruses spiked in environmental and ocean waters by targeting multiple regions specific to individual pathogen in a microarray. *Yersinia enterocolitica*,* E*. *coli*, and *S. enterica* typhimurium were identified using a microarray in the waste water with the limit of detection of 1 × 10^7^ for *S. typhimurium* cells (Maynard et al. [Link]). Inoue et al. (2015) reported the presence of 941 pathogenic bacteria in ground water and differentiated between their sources of origin. Presently, many DNA microarray‐based kits are available in the market. One such example is the phylogenetic microarray known as PhyloChip marketed by Affymetrix consisting of 500,000 oligonucleotide probes enabling detection of 8743 strains of bacteria and archaea (Nelson et al. 2011; Aw and Rose 2012).

## Next‐Generation Sequencing

Next‐generation sequencing (NGS) is an emerging and a promising tool for studying microbial diversity and prevalence in a variety of water types. NGS enables massive parallel analysis of DNA sequences from PCR amplicons and environmental nucleic acids, ensuring new dimensions for water quality assessment. Studies on waterborne microbial communities using NGS have been accomplished by targeting sequences of the hypervariable regions of small subunit (SSU) rRNA gene (e.g., V1, V3, V4 and V6 regions) and large subunit (LSU) rRNA gene (Guo et al. 2013). Genes involved in biochemical cycles (Farnelid et al. [Link]; Bowen et al. 2013) such as *nirS* (denitrification) and *nifH* (nitrogen fixation) as well as plastid SSU rRNA have been employed for NGS microbial profiling (Steven et al. 2012). Studies ushering the relationship between waterborne communities and water quality have been exploited mainly by pyrosequencing and the Illumina platform. 454 pyrosequencing (Roche) has emerged as a platform of choice (McLellan et al. 2010; Vandewalle et al. 2012) due to relatively long‐sequence read lengths of about ~1000 bp (van Dijk et al. 2014) allowing optimized sequencing conditions and bioinformatics workflows (Sergeant et al. 2012). However, Illumina (Solexa) platform allowed the read lengths of 35 bp by combining paired‐ends reads with a focus on genome sequencing (van Dijk et al. 2014) proved to be more efficient for analysis of environmental samples. NGS platforms such as Ion Torrent and single‐molecule real‐time sequencing (SMRT), for example, Pacific Biosciences, have also been applied to amplicons sequencing for profiling of microbial communities (Marshall et al. 2012; Yergeau et al. 2012). However, these technologies have not been exclusively adopted so far for studies on waterborne microbial community profiling.

## Pyrosequencing

Pyrosequencing is a technique which requires DNA polymerase, ATP sulfurylase, luciferase, and apyrase enzymes. After the incorporation of nucleotides by DNA polymerase using a single‐stranded fragment, the released pyrophosphate (PPi) is converted to ATP by ATP sulfurylase. The ATP produced derives luciferase‐mediated conversion of luciferin to oxyluciferin that in turn generates visible light signal which is detected by charged‐couple devices (CCD) sensors (Aw and Rose 2012; Siqueira et al. 2012). Pyrosequencing primarily involves concentrating the bacteria present in water and a subsequent secondary step of concentration of bacteria may be performed based on the type of bacteria present in the water sample. Then, the extracted DNA is amplified by pyrosequencing and the data obtained is analyzed by bioinformatics tools (Aw and Rose 2012; Siqueira et al. 2012). Pyrosequencing methodology has been applied to study the bacterial biofilms in drinking water distribution systems (Hong et al. 2010), mixed urban watershed (Ibekwe et al. 2013), and study the nontuberculous *Mycobacterium* communities in potable water (van der Wielen et al. 2013). Factor which limits the efficiency of this methodology is the amount of DNA which is found in water as it requires DNA at the level of picomoles, however, smaller amounts of DNA molecules may be present in water bodies. High cost, complex analysis, efficiency of data generation, and availability of massive computing power are some other factors which surmount the use of this technique (Gong et al. 2010).

## Illumina Technology

Newton et al. (2015) examined the microbial communities in waste water treatment plants and compared their occurrence in human stool samples using oligotyping of high‐throughput 16S rRNA gene sequence data. The core findings of the study revealed statistical evidences linking enrichment of specific microbial families to obesity rates in 17 U.S. cities. Recent studies suggest that application of NGS and related sequence‐based methods has proven to be an important tool for tracking human bacterial pathogens or waterborne viral stains. Monitoring bacterial pathogens in the environment would ultimately influence the strategy improvement to combat the outbreaks in a community. However, the distribution and relative representation of human bacterial pathogens significantly vary with the environmental conditions, anthropogenic impacts, and the implementation of water treatment technologies. Profiling of microbial communities in drinking water systems require collection of large amounts of water, for example, 100–2000 L (Chao et al. 2013; Shi et al. 2013; Huang et al. 2014) as the microbes in such systems tend to be low in numbers. Besides this, the yields of purified DNA obtained from drinking water systems is low requiring amplification prior to sequencing (Gomez‐Alvarez et al. 2012). Water disinfection by either chlorine or monochloramine results in selective effects on the microbial biofilms in water distribution pipelines and end‐point drinking water (Chao et al. 2013). Huang et al. (2014) investigated using 454 pyrosequencing and Illumina high‐throughput sequencing to detect possible pathogenic bacteria and virulence factors (VF) in drinking water treatment and distribution system. 16S rRNA (V3‐V4) pyrosequencing showed high bacterial diversity includingly mainly *α*‐Proteobacteria in the drinking water. Moreover, it was observed that the bacterial diversity decreased after chlorination, whereas it increased after pipeline distribution. Many pathogens were killed after chlorination, however, *P. aeruginosa* and *Leptospira interrogans* were detected in water. High‐throughput sequencing showed presence of various pathogenicity islands and virulence proteins in the drinking water, and VFs such as tanslocases, transposons, CIp proteases, and flagellar motor switch proteins were detected in the metagenomes. Sand filters are exclusively employed for purification of water around the world. However, subsequent water filtration allows microbes to form a biofilms on sand filters.

Bai et al. (2013) reported metagenomic analyses of microbial communities in sand filters used to treat groundwater for potable consumption; they observed that 90% of total microbial community was dominated by bacteria within 2 days with *α*‐Proteobacteria being the most common. Functional annotation of metagenomic datasets showed aromatic degradation pathway genes, for example, aromatic oxygenase and dehydrogenase genes, in the biofilms, envisaging potential role of microbes in the degradation of aromatic compounds in the groundwater. Cyanobacterial harmful algal blooms (cHAB) pose threat to aquatic ecosystems and render it unsafe for human consumption. The cHABs are predominantly responsible for overgrowth of cyanobacterial species many of which produce cyanotoxins such as microcystin, cylindrospermopsin, and anatoxin (Ferrão‐Filho Ada and Kozlowsky‐Suzuki 2011) and odorous compounds, for example, geosmin or 2‐methylsoborneol (Li et al. 2012). Metatranscriptomes of causative agents of cHAB, for instance, *Microcystis aeruginosa, Planktothrix agardhii*, and *Anabaena cylindrica* revealed the transcription of genes for microcystin synthesis and acquisition of nitrogen and phosphorous from the aquatic system (Steffen et al. 2015). Furthemore, NGS requires statistical methods and bioinformatic tools for the analysis of humongous amount of data generated by these methods. For instance, MOTHUR (Kozich et al. 2013) and QIIME (Caporaso et al. 2010) are commonly employed softwares for the analysis of amplicon sequences generated from Illumina technology.

## Genomics

Genomics or whole‐genome sequencing (WGS) makes use of DNA sequencing and bioinformatics tools to elucidate the function and structure of genes of both humans and pathogens. Genomics has been exclusively applied in clinical microbiology to study the infectious agents, thus, enabling comprehensive large‐scale analyses of whole genomes of pathogen. Recently, various studies have elaborated the application of WGS to trace evolution and epidemiology of microbial infections (Grad et al. 2014; Leopold et al. 2014; Petty et al. 2014). Moreover, witnessing the emergence of multidrug‐resistant infections in the new era, WGS may be deemed as an inevitable platform to track specific resistance mechanisms, including motifs on genetic elements such as plasmids and unraveling the mechanisms of gene transfer (Sivertsen et al. 2014; Stoesser et al. 2014). Mellmann et al. (2011) reported the application of whole‐genome sequencing (WGS) using the Life Technologies Ion Torrent PGM^™^ sequencer and Optical Mapping to characterize the outbreak of *E. coli* O104:H4 isolate which was held responsible for 830 cases of hemolytic uremic syndrome (HUS) and 46 deaths that occurred in Germany.

Using whole‐genome sequencing on cultured isolates from the 17 samples with pure cultures, Hasman et al. (2014) were able to obtain rapidly precise species information, and predicted antimicrobial susceptibility profiles equal to those obtained by phenotypic methods. Direct sequencing of the urine samples enabled the bacterial species identification including the detection of resistance genes. Importantly, Hasman et al. (2014) inferred that direct sequencing on the clinical samples yielded information on the presence of bacteria that were not detected using conventional (aerobic) culturing.

WGS revealed the evolution of highly pathogenic hybrid of enteroaggregative *E. coli* (EAEC) and enterohemorrhagic *E. coli* (EHEC), which emerged as outbreak clone. *E. coli* sequence type 131 (ST131) is a multidrug‐resistant (MDR) clone responsible for urinary tract and bloodstream infections. Very interestingly, the genome analyses revealed the strain ST131 to harbor virulence gene, type 1 fimbriae FimH30 allele and produce CTX‐M‐15 extended spectrum *β*‐lactamase (ESBL) to exhibit resistance to fluoroquinones. Petty et al. (2014) studied the phylogeny and molecular epidemiology of ST131 using WGS to finally unveil single lineage of ST131 found to be distinct from other extraintestinal *E. coli* strains within the B2 phylogroup. It is more evident that WGS would be the platform of choice in near future for applications such as typing, epidemiological surveillance, and outbreak investigation over other molecular methods including DNA microarray and 16S rDNA sequencing.

Notwithstanding all the technical advantages offered by WGS, there are some issues with WGS which must be addressed to make it more applicable in order to identify human pathogens. Most WGS analyses are based on the single‐nucleotide variants which are identified by comparing with the reference genome sequence. This implies that the WGS protocol must involve quality of sequencing and genome assembly and, moreover, quality and selection of reference genome (Bertels et al. 2014). In the comparative genomics, a significant phylogenetic data is excluded. So, it is imperative to study the phylogenetic data on all loci in a genome (Kwong et al. 2015). Thus, with more convenience of bioinformatics tools, the use of WGS will be more widespread. To establish a powerful epidemiological surveillance of infections, the improvements in WGS efficiency and decline in the costs are prerequisites for success of this technology in future.

## Fluorescence In Situ Hybridization (FISH)

FISH or fluorescence in situ hybridization is a cytogenetic methodology which involves hybridization between specific DNA sequences and rRNA oligonucleotide probes labeled covalently with fluorescent dyes. Fluorescence microscopy, confocal microscopy, or flow cytometry can be used to enumerate particular microbial population based on the degree of sequence complimentarity between the probe and the sample (Amann and Fuchs 2008). Characterization of microbial population in complex environments such as biofilms, quantitative study of microbial community structure in activated sludge and wastewater, detection of pathogens in water and sewage, and the mechanism of survival of pathogens have been described using the FISH technique (Amann and Fuchs 2008; Gilbride 2014). Besides this, FISH has also been applied for the detection of VBNC cells coupled with the direct viable count (DVC) assay. This assay enabled the detection of 3000 viable *E. coli*/100 mL in more than 10^**8**^ non‐*E. coli/*100 mL (Garcia‐Armisen and Servais 2004; Wingender and Flemming 2011). FISH, however, has low sensitivity and requires pre‐enrichment and concentration steps (Girones et al. 2010), which may result in inclusion of inhibitors and lead to false‐negative results (Gilbride 2014). Methods other than FISH have been efficiently used since the last decade to monitor VBNC in water bodies such as immunological techniques, RT‐PCR, and the commercial kit LIVE/DEAD^®^ *Bac*Light ^™^ assay (Barer and Bogosian 2004; Rowan 2004; Pinto et al. 2011).

## Advantages and Limitations of Rapid Methods

Rapid methods provide numerous advantages for the detection and quantification of waterborne pathogens; however, they also do possess various limitations which are summarized in Table 3.

**Table 3:** Summary of advantages and limitations of rapid methods for detection of waterborne bacteria

Method	Advantages	Limitations	References
Conventional PCR	High specificity; high sensitivity; automated	Needs purified DNA; sensitive to PCR inhibitors; cannot differentiate viable and nonviable cells	Bej et al. 1990; Tsen et al. 1998;
Multiplex PCR	High specificity; high sensitivity; automated; multiple pathogens can be detected	Primer designing is important in terms of annealing temperature; sensitive to PCR inhibitors; cannot differentiate viable and nonviable cells	Omar and Barnard (2014); Fan et al. (2008)
Real‐time PCR	High specificity; high sensitivity; multiple pathogens can be detected simultaneously; No post‐PCR processing required; can differentiate viable and nonviable cells; multiplexing is possible	Primer designing is important in terms of annealing temperature; sensitive to PCR inhibitors; cross contamination may take place	Maheux et al. 2014; Taskin et al. (2011); Li and Chen (2012)
DNA microarray	High specificity; high sensitivity; multiple pathogens' detection possible; high‐throughput	High cost; cannot differentiate between viable and nonviable cells; may involve nonspecific hybridization	Zhou 2003; Gilbride 2014; Wilson et al. (2002)
NGS	Specific and sensitive; bacterial biofilms can be characterized	Amount of DNA required is in picomoles; high cost; complex analysis; massive computing power is required	Siqueira et al. 2012; Aw and Rose 2012; Ibekwe et al. 2013;
FISH	High selectivity; can differentiate between viable and nonviable cells	Low sensitivity; requires pre‐enrichment and concentration steps for sample processing; false‐negative results possible; high cost	Amann and Fuchs 2008; Girones et al. 2010;
Immunological methods	Specific; bacterial toxins can be detected; multiple samples can be examined at a time	Low sensitivity; cross‐reactivity; false‐negative results; pre‐enrichment required to expose surface antigens	Peng and Shelef 1999; Mansfield and Forsythe 2000

PCR, polymerase chain reaction.

## Immunology‐Based Methods

Immunological methods are based on antibody–antigen interaction where a particular antibody binds to specific antigen. The method is based on the principle use of either polyclonal or monoclonal antibodies (Law et al. 2014). Immunological techniques such as enzyme‐linked immunosorbent assay (ELISA), immunofluorescence assay (IFA), and serum neutralization tests (SNTs) have been used for the detection of pathogens. In the direct immunofluorescence method, specific antibody is directly conjugated to a fluorochrome. The indirect procedure involves an additional step for the binding of the specific primary antibody to the targeted antigen, which is followed by the addition of a fluorochrome‐labeled antibody directed against the first antibody. Both ways, enumeration of fluorescently labeled cells can rapidly be achieved using epifluorescence microscopy or solid‐phase cytometry after filtration of the water sample, or by flow cytometry (Amann et al. 1990; Karo et al. 2008). Immunomagnetic separation (IMS) method can be used for the enumeration of diluted specific cells. In this method, magnetic beads coated with monoclonal or polyclonal antibodies are used which help in the purification and concentration of the targeted cells. This method has been applied to the detection of low numbers of cells in food samples (Peng and Shelef 1999; Mansfield and Forsythe 2000). In case of *E. coli* O157:H7, a monoclonal antibody specific for O157 lipopolysaccharide (LPS1) is used; however, cross reactivity with other bacteria results because of the presence of 4‐amino‐1,6‐dideoxy‐d‐mannopyranosyl, a constituent sugar of LPS (Wattam et al. 2012). The O157 antibody also binds to the LPS of *V. cholerae* O1 and *Salmonella* Typhi. Park et al. (2010) reported detection of *Salmonella* Typhimurium on an immunochromatographic strip. Colorimetry and chemiluminiscent were used as optical signal generation methods to achieve the limit of detection (LOD) of 1.8 × 10^3^ CFU/mL for *Salmonella* Typhimurium within 20 min. Several ELISA systems have been commercialized for rapid detection of *Salmonella* in raw or processed products such as TECRA *Salmonella* (Tecra International Pty Ltd, French Forest, New South Wales, Australia), *Salmonella* ELISA Test SELECTA/OPTIMA (Bioline APS, Denmark), and Vitek Immuno Diagnostic Assay System (VIDAS) (Biomerieux, Hazelwood, MO). Despite all these developments, immunological methods have got limitations such as false‐negative results, cross reactivity with closely related antigens, and low sensitivity.

## Biosensor‐Based methods

Biosensor is a self‐contained analytical device that consists of a biological recognition element (e.g., nucleic acids, microbes, enzyme and antibodies) and the transducer. The analyte (protein, toxin, peptide, vitamin, sugar, and metal ion) binds to biological element which in turn produces the electronic response that can be measured. Sometimes, the analyte is converted to a product which could be associated with the release of heat, gas, electrons, and hydrogen ions. The transducer then converts the product‐linked changes into electrical signals which can be amplified and measured (Tombelli et al. 2002; Rider et al. 2003). Transduction processes can be optical, electrochemical, mechanical, mass‐based, or magnetic; hence, in turn provides quantitative or semi‐quantitative analytical information (Koedrith et al. 2015).

**Figure 2:**
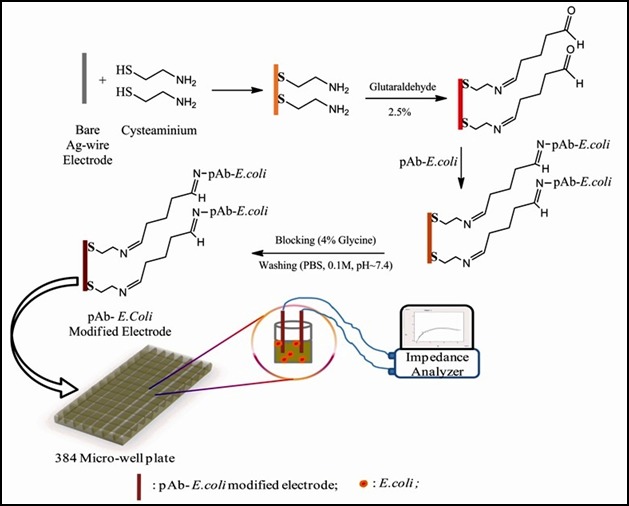
Schematic representation of Ag‐wire electrode‐based impedimetric immunosensor. Ag‐electrodes were dipped overnight in 10 mmol/L cysteaminium and dried with nitrogen stream. The pAb‐*E. coli* were coupled to carboxyl group of SAMs on modified electrode by treatment with 2.5% glutaraldehyde solution. Reminiscent aldehyde groups were blocked by exposing the modified Ag‐electrode surface to 4% glycine solution for 30 min. functionalized pAb‐*E. coli* modified Ag‐electrodes were immersed into micro well plate and connected to IVIUM CompactStat impedence analyzer. The antigen–antibody interactions at the electrode surface caused impedance change which was measured at 1–10 KHz applied frequency and 10 mV applied potential (Reproduced from Mishra et al. 2015). SAM, self‐assembled monolayers.

Optical biosensor such as surface plasma resonance biosensor, measures the change in the optical properties of the surface associated with the binding of analyte (Valadez et al. 2009). Electrochemical biosensors can be classified into various types such as amperometric, impedimetric, potentiometric, and conductometric based on the measurement of changes in current, impedance, voltage, and conductance, respectively, which is produced by analyte–bioreceptor interactions (Liao et al. 2007; Ahmed 2014). Mass‐based biosensors involve the use of piezoelectric signal which measures the small changes in mass. Binding of target pathogens (e.g., antigen) to the biological recognition element (e.g., antibodies) immobilized on the crystal causes measurable changes in the vibrational frequency of the crystal (Singh et al. 2013). Ho et al. (2004) reported optical biosensor for the detection of *E. coli* O157:H7 with fluorescent labeling where the limit of detection was 360 cell/mL. Taylor et al. (2005) used surface plasma resonance for the detection of *E. coli* O157:H7. Goat anti‐*E. coli* O157:H7 polyclonal antibodies and mouse anti‐*E. coli* O157:H7 monoclonal antibodies were used for signal amplification to obtain LOD of 10^4^ CFU/mL. Recently, Mishra et al. (2015) developed a label‐free impedimetric immunosensor for the detection of *E. coli* in environmental water samples to reach the LOD of 10^2^ CFU/mL. In this system, the silver (Ag) wire has been functionalized with the polyclonal antibody raised against *E. coli* (pAb‐*E. *coli) over self‐assembled monolayers (SAM) which form a biological transducer. Thus, binding of bacterial cell with the biological transducer brings about the change in impedance which in turn could be measured using two‐electrode system. The schematic representation of the experimental system is given in Figure 2. RAPTOR system developed by the US Naval Research Laboratory based on wave fluoroimmunoassay allows detection of waterborne pathogens simultaneously at concentrations of 5 × 10^4^ *Giardia lamblia* cysts/mL (Anderson and Rowe‐Tait 2001), 10^4^ CFU/mL *E. coli* O157:H7 (Lim 2003), 10^5^ *Cryptosporidium parvum* oocysts/mL (Kramer et al. 2007), fecal indicators such as enterococci in environmental samples at 10^5^ CFU/mL (Leskinen and Lim 2008) and 10^3^ CFU/mL *Salmonella enterica* Enteritidis (Valadez et al. 2009). Biosensors, however, do not require sample pre‐enrichment, have short analysis time and are conveniently handled unlike the nucleic acid methods which require extensive sample pre‐enrichment for the detection of pathogens. However, sensitivity to pH, mass, and temperature changes are some of the challenges which must be addressed to use these methods (Bhand et al. 2005; Ahmed 2014).

## Conclusion and Future Horizons for Water Quality Monitoring

Detection of waterborne bacteria is essential as it helps to develop strategies to quantify their numbers and obtain pathogen‐free safe water for human consumption. Conventional methods for detection of coliforms in water samples are selective but may yield false‐positive results and are laborious and time‐consuming, and moreover fail to recover VBNCs in the environmental samples. Most of the conventional methods used can merely determine presence or absence of microbes in the sample and can help in counting the cells. Rapid detection of waterborne bacteria in environmental water samples is important so as to mitigate or prevent the outbreaks of waterborne diseases and the spread of waterborne pathogens. Molecular methods such as PCR, mPCR, qPCR, and DNA microarray not only have high specificity to detect waterborne bacteria but can also differentiate between viability and nonviability of the cells as they make use of various biomarkers at the gene level. The ddPCR and qPCR are known to be highly quantitative wherein the results are obtained in the form of absolute gene copy number. However, there is a pressing need to overcome the limitations posed by PCR methods. Such limitations include the biases generated in PCR amplification due to secondary structure of the resulting amplicons, false diversity generated from sequencing errors or chimera formation, and the choice of primers used to target different small subunit rRNA hypervariable regions (Quail et al. 2012; Kozich et al. 2013; Schirmer et al. 2015). Moreover, molecular methods for either detection of microbes or quantification of nucleic acids in water circumvent some problems associated with culture‐based methods, while at the same time pose additional challenges. For instance, quantification or detection of DNA from environmental water samples is assumed to be obtained from living organisms, however, free or extraneous DNA may be detected by such methods. Thus, a prejudiced correlataion is established between the DNA copies and the cell abundance. Secondly, a sensitive and time‐efficient PCR method is the requirement of the time to detect as small as one DNA target molecule from environmental water samples. Application of NGS to the assessment of water quality is still under development and has not been broadly integrated into the epidemiological outbreak of diseases.

Microbial water quality assessment using NGS technology for detection of FIBs, pathogens, and virulence factors would herald a paradigm shift in future for establishing a correlation between the occurrence of the disease and its impact on human life. Moreover, NGS and qPCR may be coupled together for detection and quantification of nucleic acids in the environment. One such study was conducted in China by Lu et al. ([Link]) to monitor the presence of bacterial pathogens through different stages of sewage treatment plant. The metagenomic analysis showed presence of potential pathogens most closely related to *Arcobacter butzleri*,* Aeromonas hydrophila*, and *K. pneumoniae*, and the pathogenic species were subsequently confirmed by qPCR by sequencing in raw and treated sewage. NGS approaches, for example, metagenomics, metatranscriptomics, single‐cell genomics, and comparative genomics must be integrated in future to study the freshwater microbiomes to set the benchmark for real‐time water quality monitoring (Tan et al. 2015). Furthermore, investigation on water quality assessment in future should also consider the impact of emerging waterborne pathogens, that is, pathogens which have threatened the incidence of disease in humans in the last two decades and have the potentialilty to increase in future. These emerging pathogens include adenoviruses, calciviruses, coxsackieviruses, echoviruses, *Aeromonas hydrophila*,* Mycobacterium avium* and *Mycobacterium intracellulare*,* Helicobacter pylori*, cyanobacterial and other toxin‐producing freshwater algae, and microsporidia (*Enterocytozoon* and *Septata*). These pathogens must be recognized by water utilities and regulators to eunsure safe drinking and recreational water (Toranzos et al. 2007). Immunological methods such as ELISA have also been exploited for the identification of waterborne bacteria; however, these methods work well in the absence of interfering molecules such as the DNA and proteins of nontargeted cells. The major limitation of molecular and immunological methods is the need of trained personnel and costly specialized instruments. Biosensor‐based methods are rapid, easy to run, and do not require trained professionals, and these methods can be used to detect and quantify waterborne bacteria. However, the specificty and resolution of detection of biosensor technology need to be amplified for on‐site monitoring of environmental water samples. Studies on combinations of several rapid techniques might be exploited for the detection of waterborne pathogenic bacteria since a single method may not be competent enough to detect and quantify pathogens in water. Furthermore, strategies must be directed toward the development of methods effectively detecting pathogens at low concentrations; *in situ* analysis and an emphasis on multiplexing should be laid so that all closely related pathogens can be detected simultaneously.

## Conflict of Interest

None declared.
